# MIF tautomerase inhibitor TE-11 prevents inflammatory macrophage activation and glycolytic reprogramming while reducing leukocyte migration and improving Crohn’s disease-like colitis in male mice

**DOI:** 10.3389/fimmu.2025.1558079

**Published:** 2025-04-22

**Authors:** Eszter Vámos, Viola Bagóné Vántus, Péter Deák, Nikoletta Kálmán, Eva Maria Sturm, Barsha Baisakhi Nayak, Lilla Makszin, Tamás Loránd, Ferenc Jr Gallyas, Balázs Radnai

**Affiliations:** ^1^ Department of Biochemistry and Medical Chemistry, Medical School, University of Pécs, Pécs, Hungary; ^2^ Otto-Loewi Research Center for Vascular Biology, Immunology and Inflammation, Division of Pharmacology, Medical University of Graz, Graz, Austria; ^3^ Institute of Bioanalysis, Medical School, Szentágothai Research Center, University of Pécs, Pécs, Hungary

**Keywords:** Crohn’s disease, MIF, macrophage activation, M1 polarization, metabolic reprogramming

## Abstract

**Background & aims:**

Crohn’s disease (CD) is a chronic inflammatory disorder primarily affecting the gastrointestinal tract. Leukocyte recruitment, M1 macrophage polarization and associated metabolic reprogramming are hallmarks of its pathomechanism. Here, we tested TE-11, a potent MIF tautomerase inhibitor (IC_50_ = 5.63 μmol/dm^3^) in experimental Crohn’s disease in male mice, in leukocyte recruitment and in inflammatory M1 macrophage activation.

**Methods:**

2,4,6-trinitrobenzenesulfonic acid-(TNBS)-induced colitis was utilized as a CD-model in male mice. We performed macroscopic scoring and cytokine measurements. We also analyzed MIF-induced leukocyte migration and evaluated apoptosis. LPS+IFN-γ-induced RAW264.7 cells were applied as a M1 macrophage model. We performed qPCR, ROS and nitrite determinations, ELISA measurements, mitochondrial oxygen consumption rate and extracellular acidification rate determinations.

**Results:**

TE-11 improved mucosal damage, reduced inflammation score and concentration of IL-1β and IL-6 in the colon. It inhibited MIF-induced human blood eosinophil and neutrophil migration and counteracted the anti-apoptotic effect of MIF. In macrophages, MIF inhibition prevented M1 polarization by downregulating HIF-1α gene expression in LPS+IFN-γ-activated cells. Additionally, the molecule reduced mRNA transcription and protein expression of chemokine CCL-2 and cytokine IL-6 while further increasing SOD2 gene transcription and decreased ROS and nitrite production in macrophages. During inflammatory metabolic reprogramming, TE-11 prevented LPS+IFN-γ-induced metabolic shift from OXPHOS to glycolysis. Similarly to anti-inflammatory M2 cells, TE-11 improved mitochondrial energy production by increasing basal respiration, ATP production, coupling efficiency, maximal respiration and spare respiratory capacity.

**Conclusion:**

Comprehensively, TE-11, a MIF tautomerase inhibitor ameliorates CD-like colitis, reduces MIF-induced eosinophil and neutrophil migration and prevents M1 polarization and associated metabolic reprogramming; therefore, it may prove beneficial as a potential drug candidate regarding CD therapy.

## 1 Introduction

Inflammatory bowel disease (IBD) is a chronic inflammatory disorder of the gastrointestinal tract primarily affecting the intestines. Its prevalence is on the increase since more than 3.5 million patients are estimated to experience IBD in their lives just in the USA and Europe (1). IBD is manifested in two distinct forms: ulcerative colitis (UC) and Crohn’s disease (CD) (2). UC and CD both share many unpleasant symptoms, such as pain, diarrhea, weight loss and/or gastrointestinal bleeding accompanied by tissue destruction and enhanced intestinal permeability (3). Due to the compromised integrity of the intestinal epithelium, the commensal microbiota may penetrate the gut mucosa and directly activate host immune cells, such as macrophages (4).

Once activated, macrophages become polarized mainly into inflammatory M1 and anti-inflammatory M2 cells. M2 macrophages play a crucial role in the homeostasis of gut microorganisms and in regeneration of intestinal epithelium, while the M1 polarized cells instigate intestinal inflammation (5–7). M1 polarization is caused by two well characterized classical macrophage activators. On one hand, macrophages are capable of recognizing and binding specific molecules of invading microorganisms, such as lipopolysaccharides (LPS) via Toll-like receptor 4 (TLR-4) (8). The interaction between LPS and TLR-4 initiates the classical M1 macrophage activation (9). Moreover, cytokine interferon-γ (IFN-γ), which is heavily produced by lymphocytes (10) and has a pro-inflammatory role in IBD (11), also induces M1 polarization (12) through IFN-γ receptor (13). The said M1 activation is closely related to IBD (14–16), in which the cells produce large amounts of pro-inflammatory cytokines such as TNF-α, IL-6 etc. (17), reactive oxygen and nitrogen species (RONS) such as superoxide anions (18) and NO (19). Although we use the M1/M2 macrophage classification in the present work, we must emphasize the fact that this is an oversimplified model and macrophage polarization encompasses a much broader, context-dependent spectrum (12, 20).

M1 polarization, which can also be mediated by non-coding RNAs (21), represents a complete reprogramming of cellular metabolism, during which cells adapt to new conditions (22). LPS- or IFN-γ-stimulated macrophages show an altered metabolic phenotype referred to as Warburg metabolism (23, 24). The Warburg effect is characterized by switching the cellular energy metabolism from oxidative phosphorylation (OXPHOS) to aerobic glycolysis with lactate production despite sufficient oxygenation of the tissue (25). The enzyme lactate dehydrogenase synthesizes lactate from the glycolytic end product pyruvate by simultaneously oxidizing NADH to NAD^+^ to maintain glycolysis (26), a less efficient way of ATP synthesis. On the contrary, OXPHOS is a more efficient process for ATP generation. It occurs in the mitochondrial inner membrane, where NADH and succinate reduce complex I (CI) and CII of the electron transport chain (ETC), respectively. CI, CIII and CIV pump protons across the membrane and the resulted proton motive force drives the ATP synthesis via F_O_F_1_-ATP-synthase (27). Additionally, electrons passing across the ETC reduce O_2_ via protons to H_2_O in CIV. This latter process accounts for the vast majority of mitochondrial oxygen consumption (27). Thus, in Warburg metabolism, cells are forced to utilize higher amounts of glucose to produce ATP and lactate since the energetically more effective mitochondrial OXPHOS is downregulated (25, 28). Interestingly, despite a decreased flux through TCA cycle, mitochondrial succinate oxidation by succinate dehydrogenase (TCA enzyme) increases in macrophages, in which the combination with higher mitochondrial membrane potential enhances ROS production and stimulates M1 activation. In conclusion, M1 macrophages re-utilize their mitochondria from OXPHOS to ROS production, thereby promoting M1 polarization and induce inflammation (29). Interestingly, macrophages undergoing Warburg-type metabolism were found trapped in the inflammatory M1 condition. They were unable to undergo M1 to M2 polarization following IL-4 treatment, which is a strong activator of M2 polarization in macrophages (30, 31). Furthermore, sufficient mitochondrial function appears to be essential for the stimulation of M1 to M2 polarization (32). Thus, both inhibiting glycolytic reprogramming and improving mitochondrial function may prevent M1 macrophage activation thereby reducing inflammation.

The cytokine macrophage migration inhibitory factor (MIF) promotes M1 macrophage polarization (33–35) and has long been implicated in the pathomechanism of IBD (36, 37). For example, plasma MIF levels are enhanced in both CD and UC patients when compared to healthy individuals. Neutralizing anti-MIF antibodies improve the clinical manifestations of CD-like, 2,4,6-trinitrobenzenesulfonic acid-(TNBS)-induced colitis and UC-like dextran sulphate sodium-(DSS)-induced experimental colitis in mice (38). Additionally, MIF deficient mice are resistant against colitis, while reconstitution of MIF^-/-^ knock out mice with MIF^+/+^ immune cells increased colitis severity (36). MIF has several enzymatic activities such as the enigmatic tautomerase and MIF tautomerase inhibitors improved DSS-induced murine colitis (39) and TNBS-colitis in rats (40). Surprisingly, despite the above results, little is known in reference to the role of MIF tautomerase activity regarding macrophage metabolic reprogramming and mitochondrial dysfunction during M1 activation in colitis.

**Figure 1:**
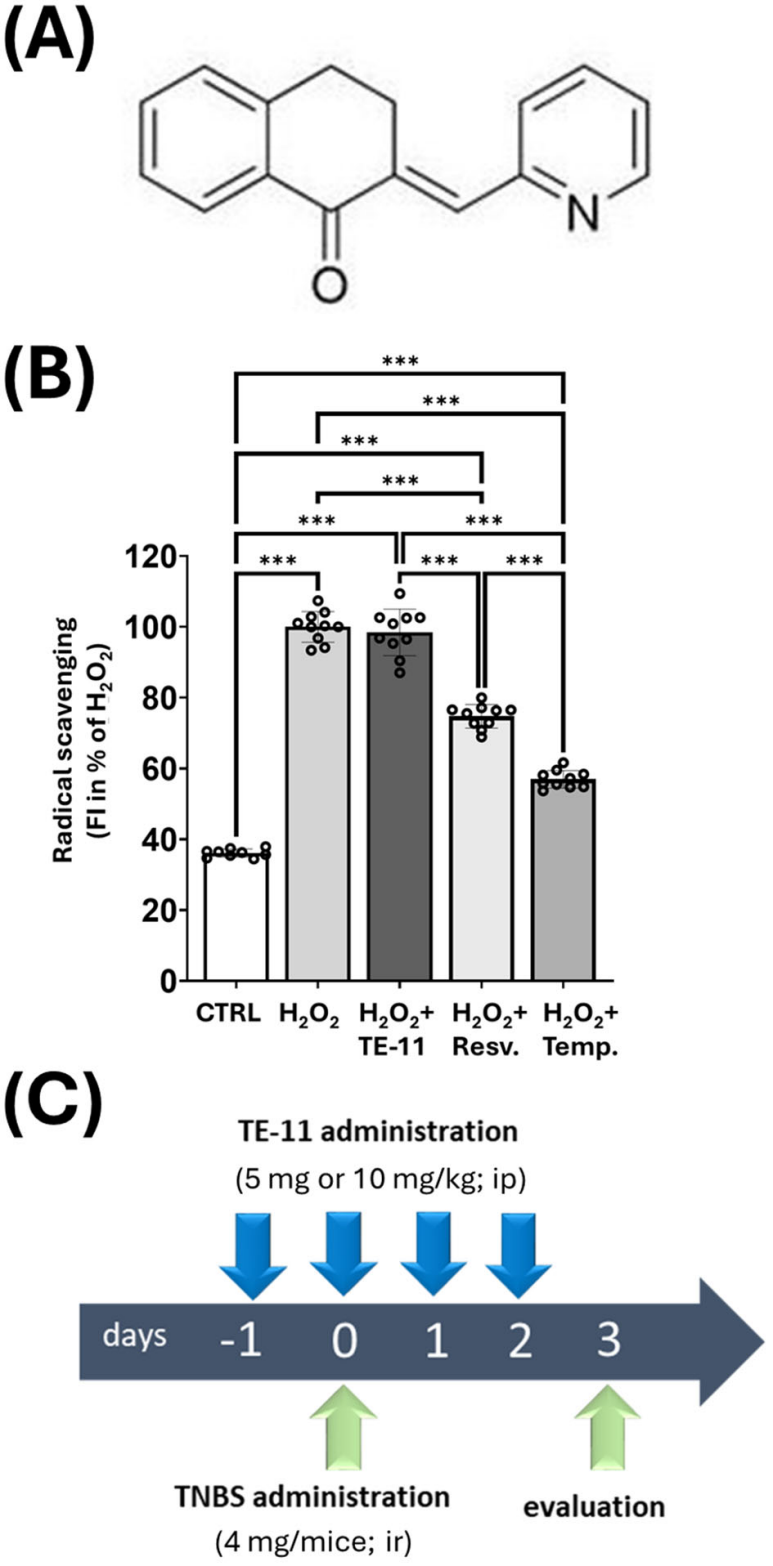
Structural formula and antioxidant effect of TE-11 in a cell free system, and experimental model of TNBS-induced colitis: **(A)** Chemical structure of (2E)-2-(pyridin-2-ylmethylidene)-3,4-dihydronaphthalen-1-one **(B)** Oxidation of DHR1,2,3 was induced by 100 μM H_2_O_2_ in the presence of TE-11, resveratrol or tempol. Data are presented as means ± SD (n = 10) One-way ANOVA ***P < 0.001 **(C)** Timescale and treatments in TNBS colitis model. *Resv, resveratrol; Temp, tempol; TNBS, 2,4,6-trinitrobenzene sulfonic acid; ip, intraperitoneal; ir, intrarectal*.

In the present study, we investigated whether TE-11 (**Figure 1A**), a new pharmacological MIF tautomerase inhibitor, which has no radical scavenging effect (**Figure 1B**), and it is not cytotoxic in the applied concentration (**Supplementary Figure S1**), but effectively prevents both ketonase (IC_50_ = 5.63 μmol/dm^3^) and enolase (IC_50_ = 28.58 μmol/dm^3^) sub-activities of MIF tautomerase (41) has a beneficial effect in a CD-like colitis among mice. Since macrophages are deeply involved in the pathomechanism of IBD, we investigated many aspects of M1 macrophage activation including RONS and inflammatory cytokine production, glycolytic activity and mitochondrial energy metabolism in LPS+IFN-γ-induced RAW264.7 cells. We also assessed the effect of TE-11 upon leukocyte migration using isolated human blood eosinophils and neutrophils.

## 2 Results

### 2.1 TE-11 improves TNBS-induced colitis in mice

**Figure 2:**
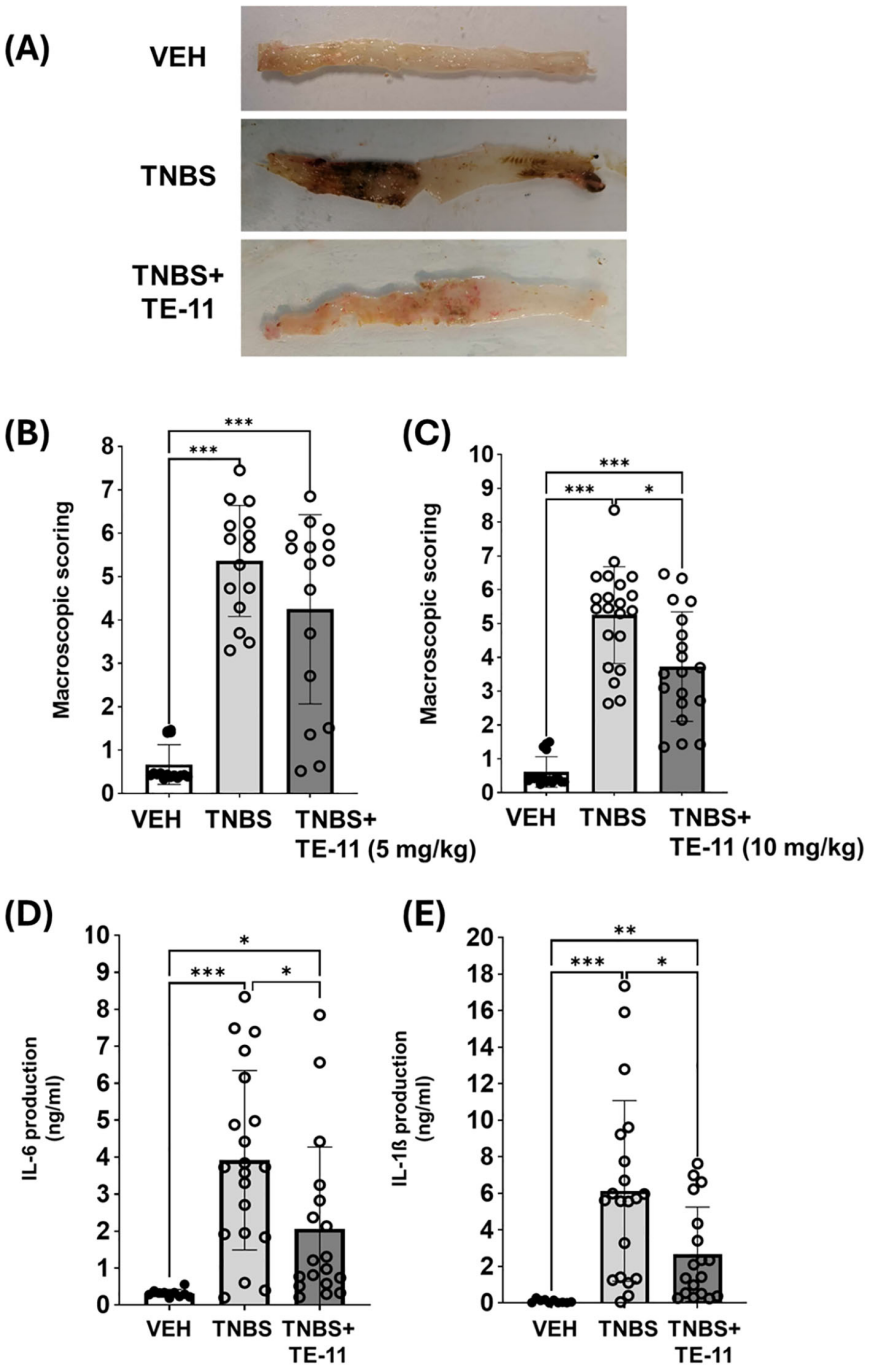
TE-11 treatment improves TNBS-induced colitis in mice. **(A)** Representative images of the colon **(B)** Macroscopic scores (5 mg/kg TE-11), data were combined from 2 separate experiments (n=15-16). **(C)** Macroscopic scores (10 mg/kg TE-11), data were combined from 3 separate experiments (n=17-21) **(D)** IL-6 and **(E)** IL-1β concentrations were measured with ELISA-kits (optical density, 450 nm). Data are presented as means+SD (combined data of 3 separate experiments (n=10-20). Kruskal-Wallis H-test **(B, C)** and Welch ANOVA **(D, E)** *P < 0.05, **P < 0.01, ***P < 0.001. *VEH, vehicle; TNBS, 2,4,6-trinitrobenzene sulphonic acid*.

To evaluate the effect of TE-11 in a mouse model of CD (42), TNBS-colitis was applied (**Figure 1C**). One bolus of TNBS-induced severe tissue destruction in the colon characterized by hemorrhagic ulcerations (**Figure 2A**) and high macroscopic scores (**Figures 2B, C**). TE-11 at a dose of 10 mg/kg reduced tissue damage primarily by reducing the ulcer size (**Figure 2A**) and resulted in a decreased inflammation score in comparison to the TNBS group (**Figure 2C**). TNBS also induced proinflammatory cytokine production in the colon (**Figures 2D, E**). We measured enhanced levels of IL-6 (**Figure 2D**) and IL-1β (**Figure 2E**) in tissue extracts, which were markedly reduced due to the TE-11 treatment.

### 2.2 TE-11 attenuates human neutrophil and eosinophil migration induced by MIF, IL-8 or CCL11

**Figure 3:**
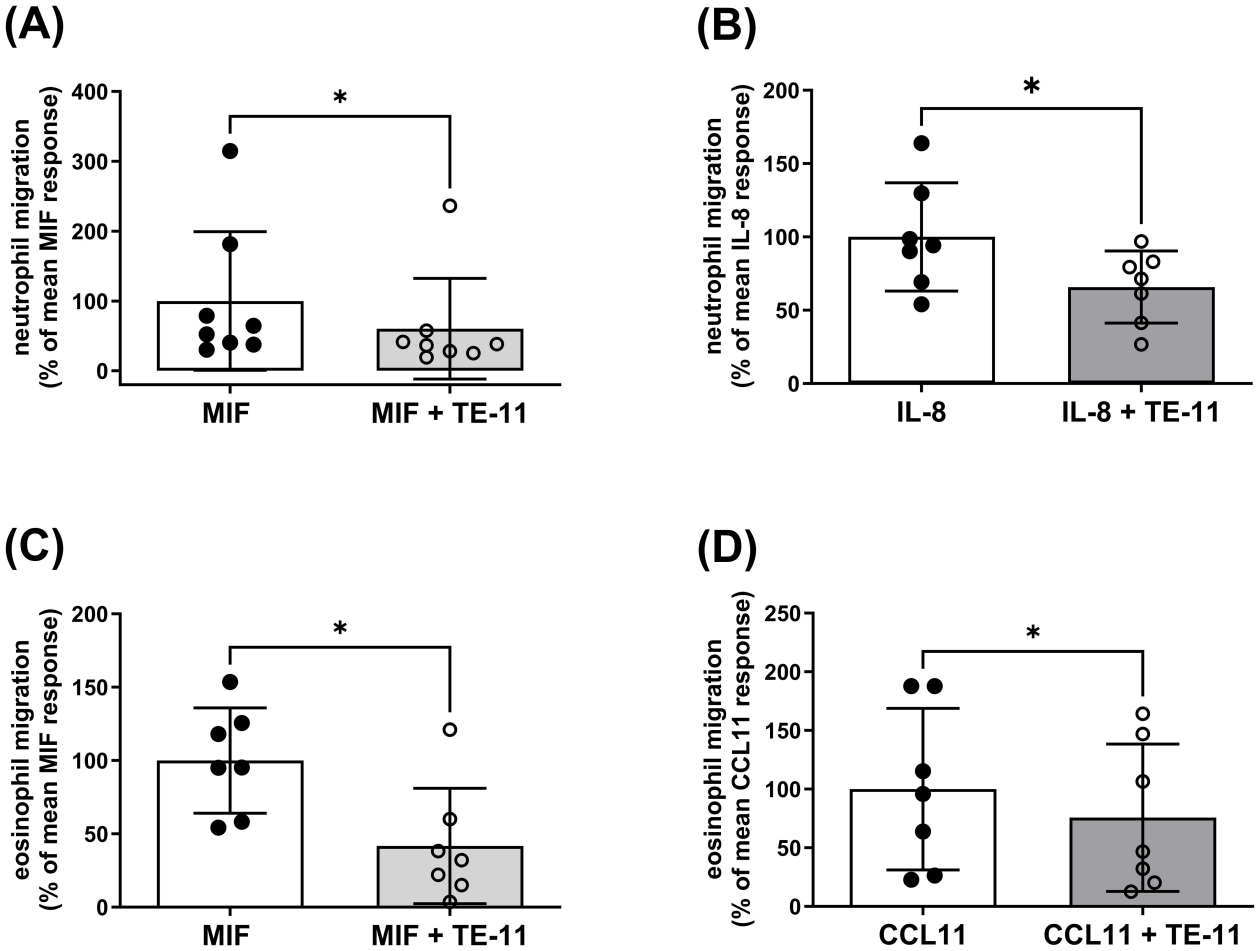
TE-11 alleviates the migratory responsiveness of human neutrophils and eosinophils towards MIF and major chemoattractants. **(A, B)** Polymorphonuclear leukocytes (PMNL) and **(C, D)** purified eosinophils were pretreated with TE-11 (20 µM) at 37°C for 30 min and were allowed to migrate towards **(A, C)** MIF (3 nM, n = 7-8), **(B)** IL-8 (10 nM, n = 6) or **(D)** CCL11 (10 nM, n = 7) in a micro-Boyden chamber at 37°C for 60 min. Migrated cells were enumerated by flow cytometry on a BD Canto II flow cytometer (acquisition set for 30 sec at medium flow rate) and expressed as % of the respective mean control response (MIF, IL-8 or CCL11 alone). Data are shown as mean ± SD of indicated independent experiments. Paired t-test or Wilcoxon test, *P < 0.05. All experiments were performed in technical triplicates. *MIF, macrophage migration inhibitory factor*.

To investigate the effect of TE-11 on leukocyte migration, purified human peripheral blood eosinophils or isolated polymorphonuclear leukocytes, mainly comprising neutrophils, were pretreated with TE-11 (20 µM) or vehicle for 30 min prior to the assay (**Figure 3**). Since IL-8 is a specific neutrophil (43) and CCL11 is a specific eosinophil (44) chemotactic factor, neutrophil migration was induced by MIF (**Figure 3A**) or IL-8 (**Figure 3B**), while eosinophil migration was stimulated by MIF (**Figure 3C**) or CCL11 (**Figure 3D**). Data were analyzed by flow cytometry (**Supplementary Figure S2**) and expressed as percent of MIF, IL-8 or CCL11 responses, respectively. We observed that TE-11 reduced MIF-stimulated neutrophil migration by 40% and IL-8-stimulated responses by 34% (**Figures 3A, B**). Similarly, TE-11 attenuated eosinophil migration towards MIF by 55% and CCL11-induced migration by 24% (**Figures 3C, D**).

### 2.3 TE-11 prevents the anti-apoptotic effect of MIF in isolated human neutrophils and eosinophils

**Figure 4:**
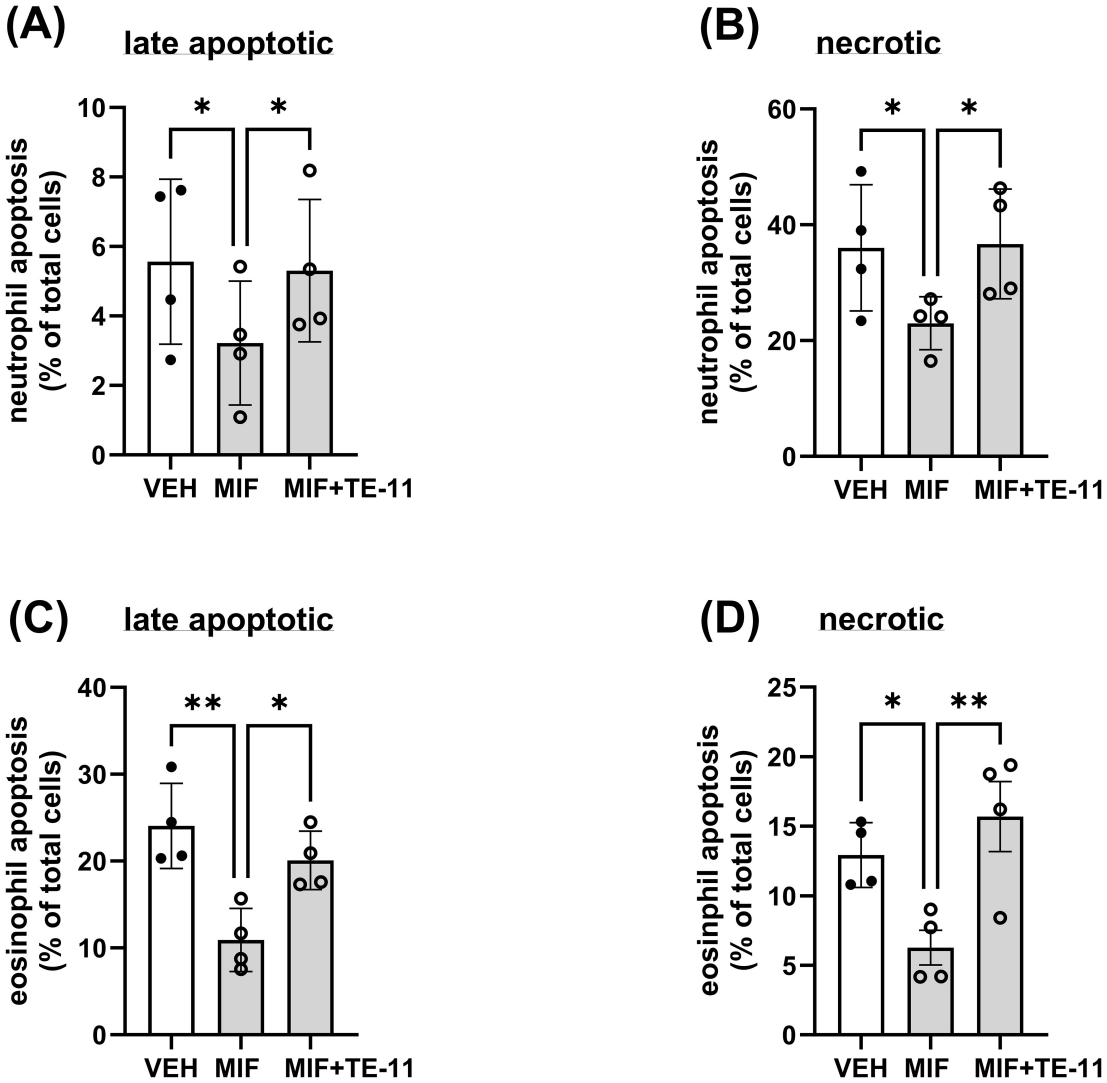
TE-11 counteracts the anti-apoptotic effect of MIF in human neutrophils and eosinophils. **(A, B)** Polymorphonuclear leukocytes (PMNL, n=4) and **(C, D)** purified eosinophils (n=4) were pretreated with TE-11 (20 µM) at 37°C for 60 min in RPMI 1640 medium supplemented with 1% FBS and 1% Penicillin/Streptomycin. Afterwards, MIF (500 nM) or vehicle control (PBS+BSA) was added to the cells. After 24 hours, cells were stained with APC-annexin-V (1/100) and Propidium iodide (1/50). Samples were immediately analyzed on a BD Canto II flow cytometer (acquisition set for 60 sec at medium flow rate). Data of **(A, C)** late apoptotic cells (annexin-V positive/PI positive) and **(B, D)** necrotic cells (annexin-V negative/PI positive) cells are presented. Data are shown as mean ± SD of indicated independent experiments and expressed as % of the vehicle control (RPMI 1640 only). One-way ANOVA, *P < 0.05, **P < 0.01. All experiments were performed in technical triplicates. *VEH, vehicle; MIF, macrophage migration inhibitory factor*.

MIF has been demonstrated to inhibit neutrophil apoptosis through diverse mechanisms (45, 46). Thus, we investigated the effect of TE-11 on neutrophil and eosinophil cell death. MIF decreased late apoptosis (**Figure 4A**) and necrosis (**Figure 4B**) in neutrophils and eosinophils (**Figures 4C, D**). TE-11 pretreatment significantly increased the amount of apoptotic and necrotic neutrophils (**Figures 4A, B**) and eosinophils (**Figures 4C, D**) when compared to MIF-treated cells.

### 2.4 MIF inhibition decreases HIF-1α mRNA transcription and protein expression in macrophages

**Figure 5:**
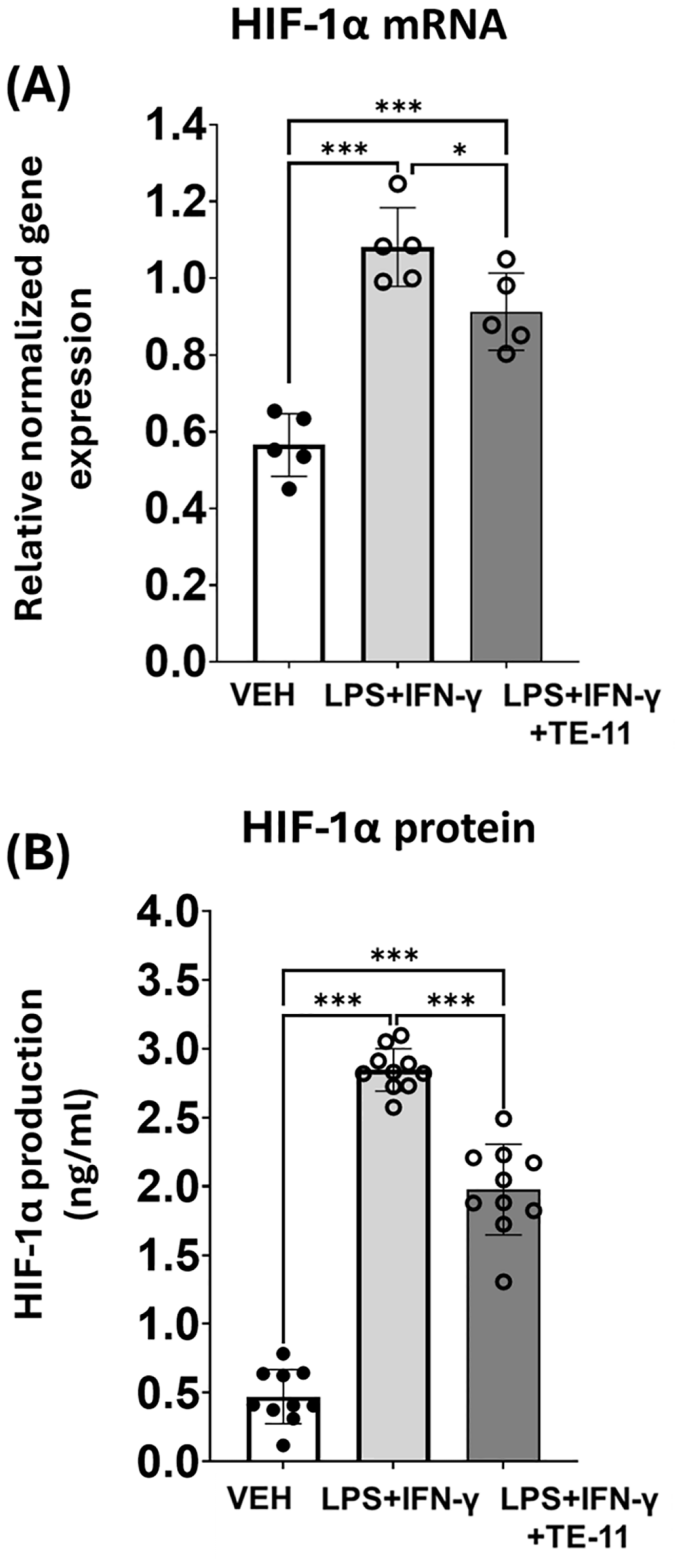
TE-11 decreased HIF-1α mRNA transcription and protein expression in activated macrophage cells. **(A)**: HIF-1α mRNA transcription and **(B)** protein expression, measured from the supernatant of the treated cells. Data (combined from 5 separate experiments with 1 or 2 parallels n=5) are expressed as mean ± SD, Welch ANOVA **(A)** and One-way ANOVA **(B)** *P <0.05, ***P < 0.001. *LPS, lipopolysaccharide; IFN-γ, interferon-γ*.

LPS+IFN-γ treatment induced HIF-1α mRNA transcription (**Figure 5A**) and protein expression as was measured in RAW264.7 cell supernatants (**Figure 5B**). TE-11 treatment inhibited both the mRNA transcription (**Figure 5A**) and protein translation of HIF-1α (**Figure 5B**).

### 2.5 TE-11 modulates inflammatory mRNA transcription in macrophages

**Figure 6:**
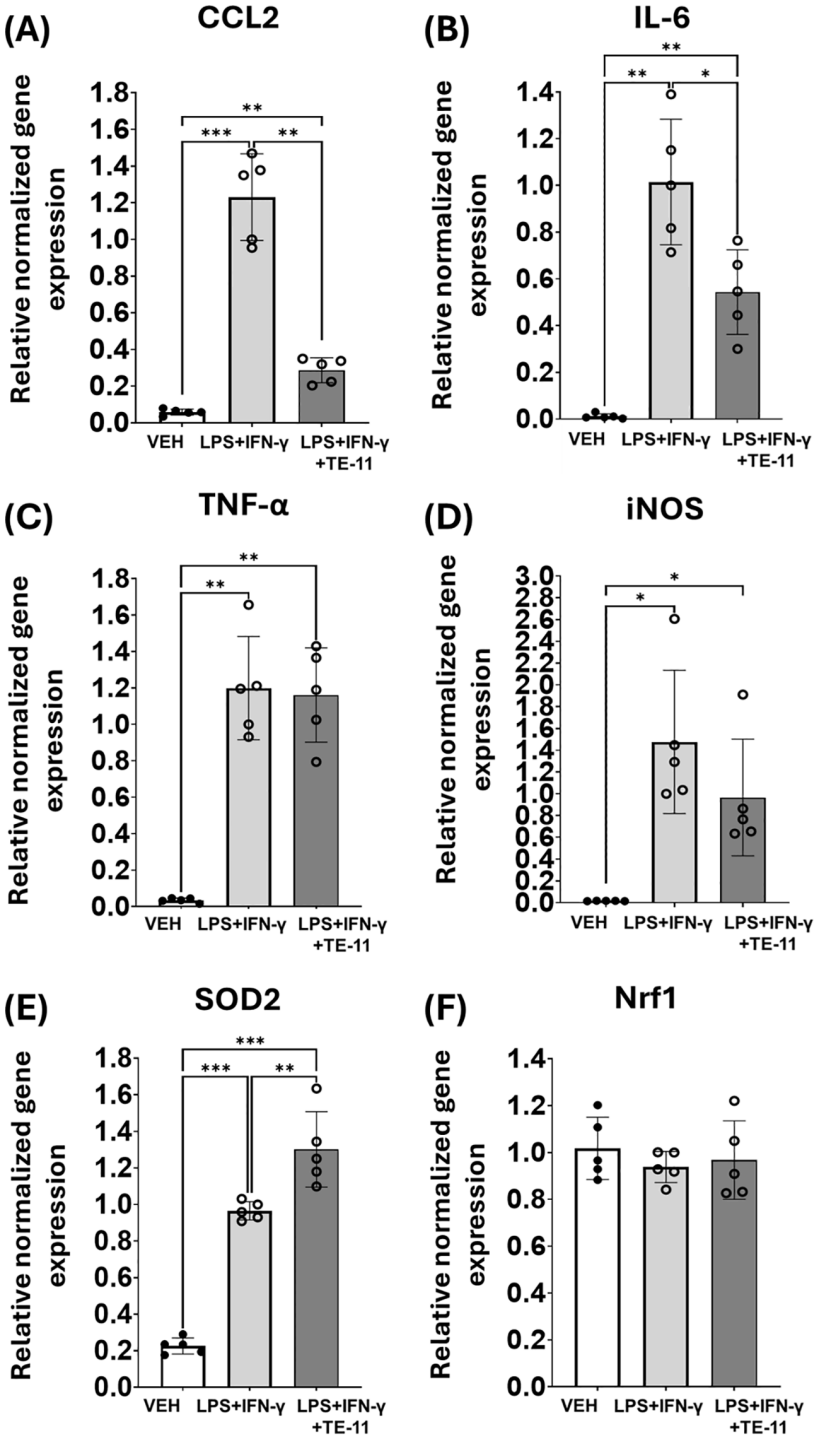
TE-11 decreased CCL2 and IL-6, while it increased SOD2 mRNA transcription in activated macrophage cells. RAW264.7 cells were pretreated with 20 μM TE-11 for 30 minutes. Then, macrophages were treated with LPS (0.1 μg/ml) + IFN-γ (0.01 μg/ml) for 8 hours. VEH and LPS+IFN-γ groups received the same amount of DMSO as TE-11-treated cells. **(A)** CCL2, **(B)** IL-6, **(C)** TNF- α, **(D)** iNOS, **(E)** SOD2, **(F)** Nrf1 relative normalized gene expressions are shown. Data (combined from 5 separate experiments n=5) are expressed as mean ± SD, Welch ANOVA **(D–F)** and One-way ANOVA **(A–C)** *P <0.05, **P < 0.01, ***P < 0.001. *LPS, lipopolysaccharide; IFN-γ, interferon-γ*.

LPS+IFN-γ treatment induced the mRNA transcription of numerous inflammatory genes in macrophages (**Figure 6**). We found elevated levels of CCL2 (**Figure 6A**), IL-6 (**Figure 6B**), TNF-α (**Figure 6C**), iNOS (**Figure 6D**) and SOD2 (**Figure 6E**) gene transcripts compared to the VEH group. In contrast, TE-11 inhibited CCL2 (**Figure 6A**) and IL-6 (**Figure 6B**) mRNA transcription, while it failed to modulate TNF-α (**Figure 6C**) and iNOS gene transcription (**Figure 6D**). Additionally, TE-11 further increased the transcription of the SOD2 (**Figure 6E**) gene in comparison to LPS+IFN-γ-activated cells. There were no alterations detected in Nrf1 mRNA transcription in our model.

### 2.6 TE-11 inhibited proinflammatory cytokine and RONS production in M1 activated macrophages without a direct antioxidative effect

**Figure 7:**
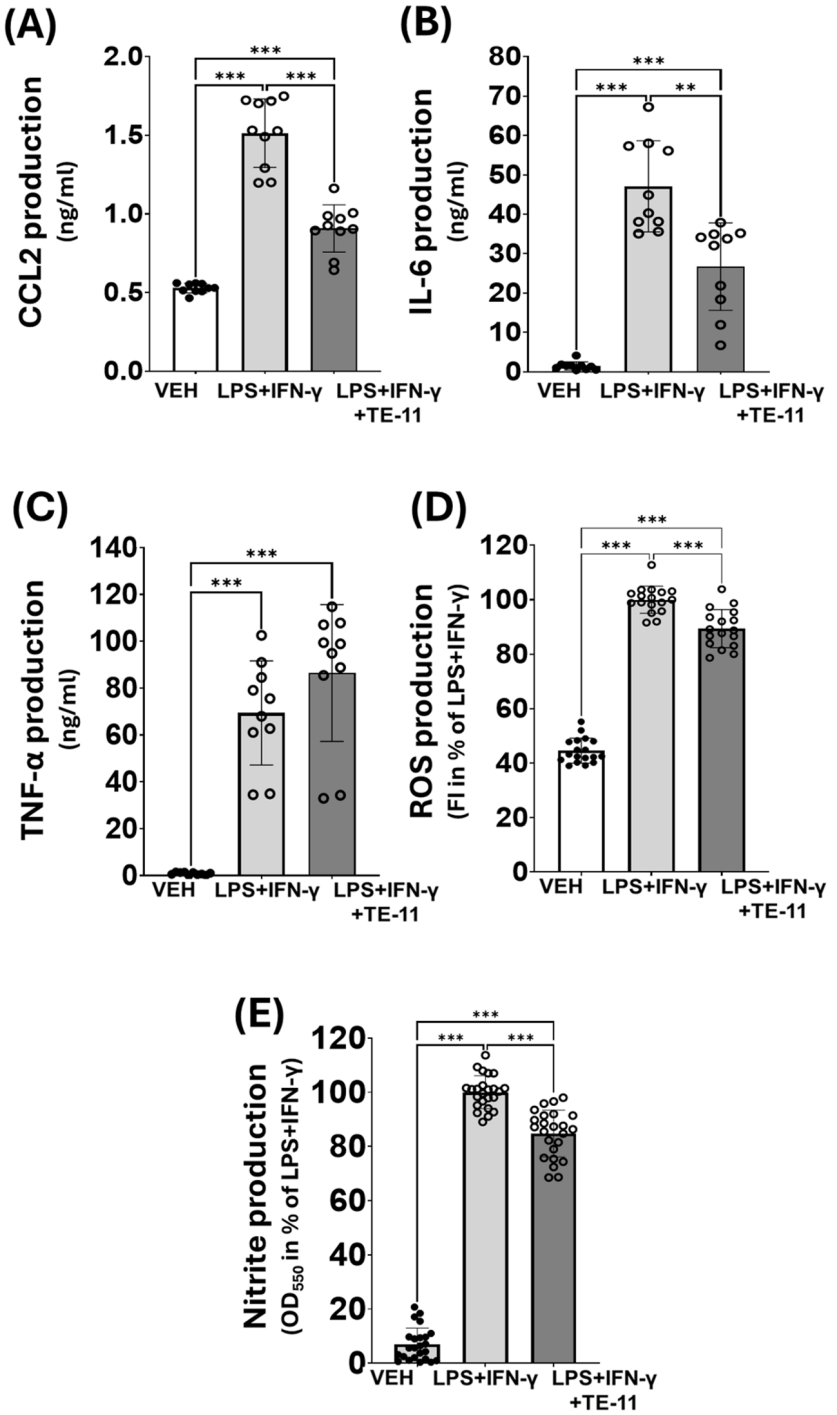
TE-11 inhibited CCL-2, IL-6, ROS and nitrite production in activated macrophages. **(A)** CCL2 **(B)** IL-6 and **(C)** TNF- α production. Combined data of n=10 [results of 5 independent experiments with 2 parallel measurements]. **(D)** ROS, **(E)** Nitrite production. Combined data of n=18 [results of 3 independent experiments with 6 parallel measurements]. Results are presented as means+SD. Welch ANOVA **(A-D)** and One-way ANOVA **(E)** **P < 0.01, ***P<0.001. *VEH, vehicle; LPS, lipopolysaccharide; IFN-γ, interferon-gamma*.

We tested the radical scavenging effect of TE-11 in a cell-free system (47). EDTA-Fe^2+^ catalyzed the decomposition reaction of H_2_O_2,_ resulting in increased levels of hydroxyl radicals when compared to CTRL. TE-11 treatment failed to reduce the concentration of the hydroxyl radicals, while two known radical scavengers, resveratrol (48) and tempol (49) significantly decreased it (**Figure 1B**). We equally tested TE-11 on RONS and cytokine production of LPS+IFN-γ-induced RAW264.7 cells. LPS+IFN-γ induced CCL2 (**Figure 7A**), IL-6 (**Figure 7B**), TNF-α (**Figure 7C**), ROS (**Figure 6D**) and nitrite (**Figure 6E**) production in macrophages. Our results revealed TE-11 diminishes CCL2, IL-6, ROS and nitrite levels, however, failed to modulate TNF-α amounts when compared to VEH cells (**Figure 7**). For IL-6 and nitrite, we used two additional concentrations of TE-11 and found that the inhibitory effect on IL-6 and nitrite production was the most prominent at a 20 μM concentration (**Supplementary Figure S3**).

### 2.7 The MIF tautomerase inhibitor TE-11 reduced basal extracellular acidification rate in RAW264.7 cells

**Figure 8:**
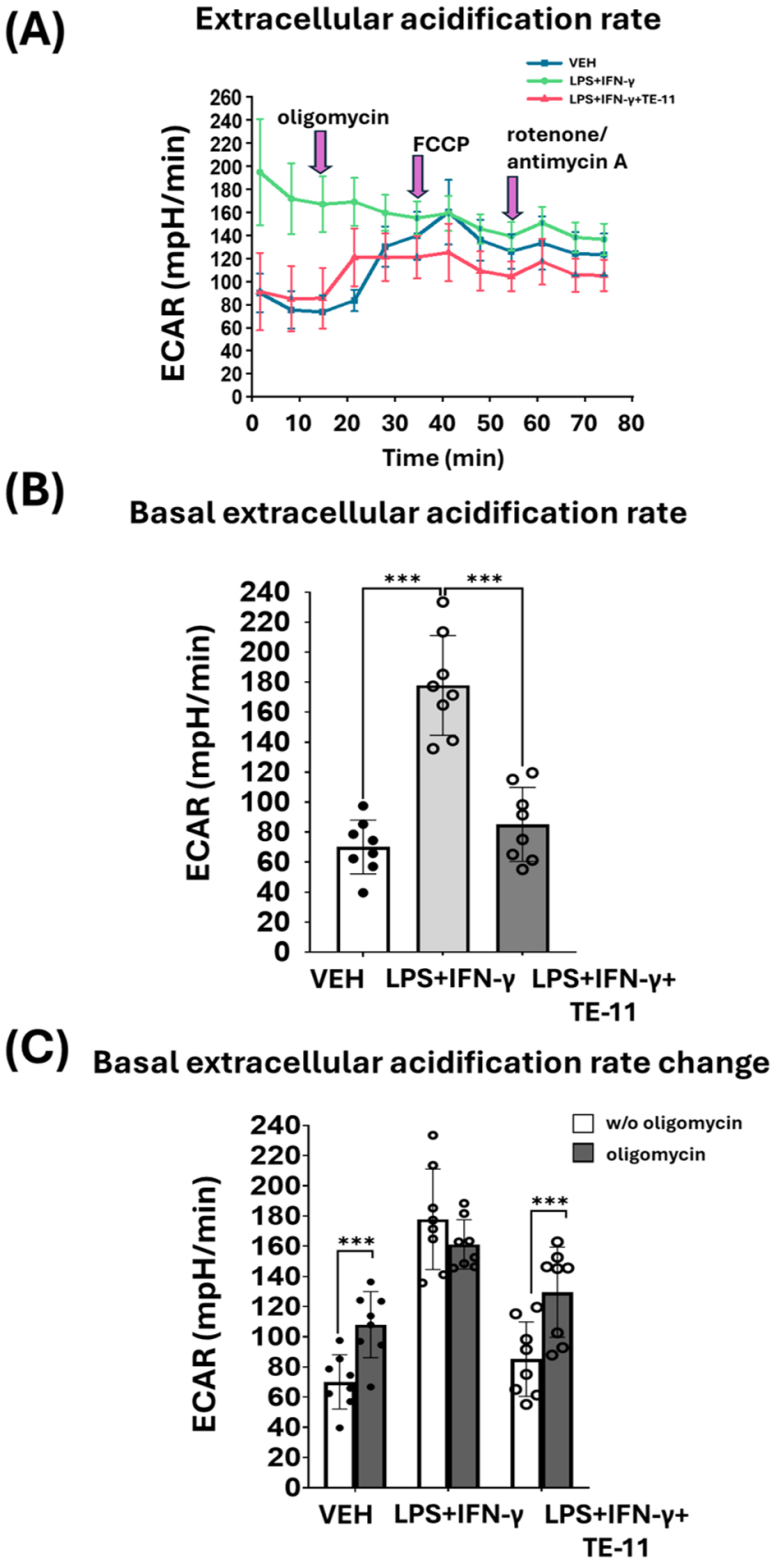
TE-11 diminished aerobic glycolysis in activated macrophages. RAW264.7cells were pretreated with 20 μM TE-11 and then induced with LPS (0.1 μg/ml) + IFN-γ (0.01 μg/ml) for 8 hrs. After the treatment Seahorse XFp Mito Stress test was performed. VEH and LPS+IFN-γ groups received the same amount of DMSO as the TE-11-treated cells. During the measurement oligomycin, FCCP, and the mixture of rotenone and antimycin A were added. The final concentrations of the inhibitors and uncoupling agent were 1 μM. **(A)** Extracellular acidification rate (ECAR), **(B)** basal ECAR, and **(C)** ECAR changes are shown. ECAR changes were determined by the difference between ECAR values before and after oligomycin injection. Data are presented as means ± SD (combined data of n=8 [results of 4 independent experiments with 2 parallel measurements]) One-way ANOVA and paired samples *t*-test ***P < 0.001. *ECAR, extracellular acidification rate; VEH, vehicle LPS; lipopolysaccharide; IFN-γ, interferon-γ*.

M1 polarized macrophages switch their energy metabolism from OXPHOS to aerobic glycolysis. Therefore, we analyzed glycolytic activity by measuring ECAR in activated macrophages. LPS+IFN-γ induced a marked increase, while TE-11 caused a significant reduction in basal ECAR in comparison with the LPS+IFN-γ-treated cells (**Figure 8A** [1-3 points of the measurement] and **Figure 8B**). Oligomycin enhanced ECAR in VEH and LPS+IFN-γ+TE-11 groups, however, failed to increase its level in the LPS+IFN-γ-treated cells (**Figure 8A** [4-6 points of the measurement] and **Figure 8C**). FCCP (**Figure 8A** [7-9 points of the measurement]) and rotenone plus antimycin A (**Figure 8A** [10-12 points of the measurement]) did not significantly affect ECAR in either treatment groups. We used two additional concentrations of TE-11 (5 μM, 10 μM) for ECAR measurement and found that the inhibitory effect on ECAR at the lower concentrations was comparable to that at 20 μM (**Supplementary Figure S4**).

### 2.8 TE-11 improved mitochondrial respiration, ATP production and coupling efficiency

**Figure 9:**
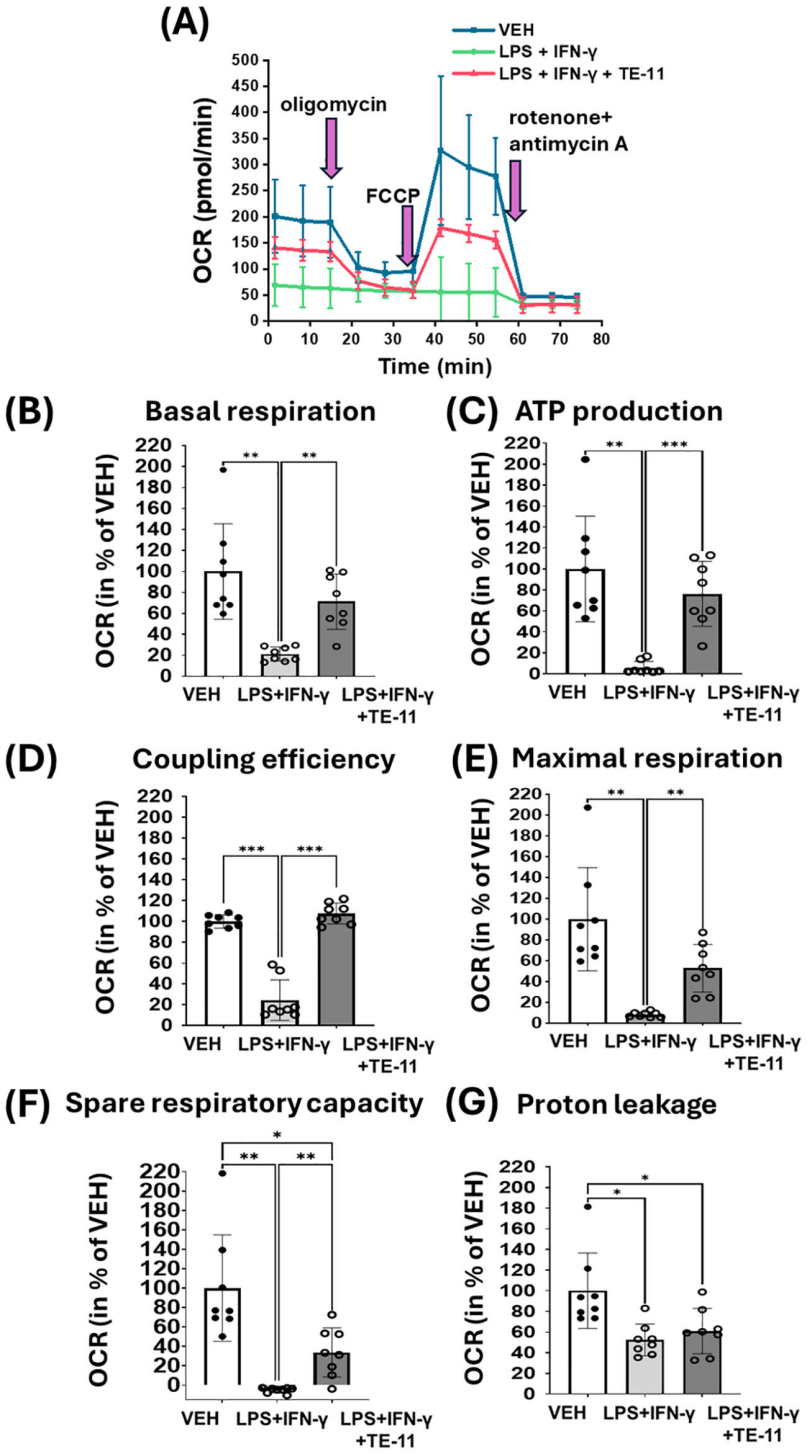
TE-11 protected mitochondrial respiration and improved mitochondrial bioenergetic parameters in activated macrophages. RAW264.7 cells were pretreated with 20 μM TE-11 for 30 minutes. Then macrophages were treated with LPS (0.1 μg/ml) + IFN-γ (0.01 μg/ml) for 8 hours. VEH and LPS+IFN-γ groups received the same amount of DMSO as the TE-11-treated cells. Oligomycin, FCCP and the mixture of rotenone and antimycin A were added sequentially during the measurement in a final concentration of 1 μM. **(A)** Measurement of oxygen consumption rate. **(B)** Basal respiration, **(C)** ATP production, **(D)** Coupling efficiency **(E)** Maximal respiration, **(F)** Spare respiratory capacity, and **(G)** Proton leakage were determined. Results (data combined from 4 separate experiments with 2 parallel measurements, n=8) are expressed as mean+SD. Welch ANOVA **(A-C, E-G)** and One-way ANOVA **(D)** *P < 0.05, **P < 0.01, ***P < 0.001. *OCR, oxygen consumption rate; VEH, vehicle; LPS, lipopolysaccharide; IFN-γ, interferon-γ*.

M1 polarized macrophages downregulate mitochondrial OXPHOS. Therefore, we analyzed the effect of TE-11 on mitochondrial oxygen consumption in LPS+IFN-γ-treated RAW264.7 cells (**Figure 9A**). By using oligomycin, FCCP and rotenone plus antimycin A, many further aspects of the mitochondrial energy status were measured and calculated (**Figure 9B-G**). LPS+IFN-γ treatment strongly attenuated mitochondrial OXPHOS by reducing basal respiration (**Figure 9A** [1-3 points of the measurement] and **Figure 9B**). Oligomycin reduced (**Figure 9A** [4-6 points of the measurement]) and FCCP (**Figure 9A** [7-9 points of the measurement]) enhanced basal respiration in VEH and LPS+IFN-γ+TE-11 treatment groups, however, they failed to modulate it in the LPS+IFN-γ-treated cells. LPS+IFN-γ increased ATP production (**Figure 9C**), coupling efficiency (**Figure 9D**), maximal respiration (**Figure 9E**), spare respiratory capacity (**Figure 9F**) and proton leakage (**Figure 9G**). TE-11 effectively improved all enlisted parameters with the exception of proton leakage. We used two additional concentrations of TE-11 (5 μM, 10 μM) for OCR measurement and found that the improvement in OCR and bioenergetic parameters was the most prominent at a concentration of 20 μM. (**Supplementary Figure S5**).

## 3 Discussion

A very recent pioneering study strongly supports the fact that macrophage activation and metabolic reprogramming play a fundamental role in the development of IBD in a shocking number of IBD patients (50). In our present report, we demonstrated how TE-11, a novel and potent MIF tautomerase inhibitor, attenuated inflammatory macrophage activation and associated metabolic shift from OXPHOS to aerobic glycolysis. Additionally, TE-11 reduced leukocyte migration and ameliorated Crohn’s diseases such as experimental colitis in mice.

First, we investigated whether the MIF tautomerase inhibitor TE-11 (**Figure 1A**) may improve CD-like experimental colitis in mice. Therefore, we applied TNBS-colitis (**Figure 2B**) since many of the symptoms, histological and biochemical characteristics of this model are similar to human CD (42). Additionally, the similarity between CD and TNBS-colitis is an increase in MIF expression, of which, was equally observed among CD patients (51) and in the colon of TNBS-treated rodents (52, 53). TE-11 alleviated the severity of colitis in mice (**Figures 2A, B**), which was primarily characterized by a strong reduction in ulcer size. The decrease in inflammation scores was accompanied by a significant decline in the tissue expression of IL-6 and IL-1β (**Figures 2C, D**).

In IBD, the proinflammatory cytokines IL-6 and IL-1β are mainly primarily produced by leukocytes which are recruited into the colon. Leukocyte recruitment, however, is largely regulated by MIF (54). Therefore, we investigated whether TE-11 may inhibit leukocyte migration (**Figure 3**). In addition to MIF, IL-8, a specific neutrophil (43) and CCL11, a specific eosinophil (44) chemotactic factor were utilized to induce migratory responses. As a cytokine, MIF binds to its receptors CD74 (55, 56) and CXCR2 (54) and stimulates chemotaxis. TE-11 reduced MIF-induced neutrophil (**Figure 3A**) and eosinophil (**Figure 3B**) migration, possibly due to its ability to bind to the tautomerase catalytic domain of MIF (41), which is responsible for protein/receptor interactions (57). This implies blocking the tautomerase catalytic domain by TE-11 prevents MIF receptor binding, thereby attenuating chemotaxis. Surprisingly, MIF tautomerase inhibition equally blocked IL-8-induced neutrophil migration (**Figure 3B**). A possible explanation for this phenomenon suggests IL-8-induced neutrophils have been shown to express MIF (58), which can further enhance leukocyte migration. Accordingly, TE-11 may inhibit this secondary, MIF-induced migration rather than the direct IL-8-induced neutrophil chemotaxis. TE-11 also slightly reduced CCL11-induced eosinophil migration (**Figure 3D**). This likely is the result due to the recognized cross-talk between MIF and CCL11 in eosinophils (59), i.e., blocking MIF receptor binding prevents MIF signaling and moderates the effect of CCL11. Furthermore, MIF inhibited late apoptotic and necrotic cell death in neutrophils (**Figure 4A, B**) and eosinophils (**Figures 4C, D**), which was significantly counteracted by TE-11 treatment (**Figure 4**) and can be satisfactorily explained by the prevention of receptor activation by TE-11. Comprehensively, TE-11 effectively inhibited MIF-induced leukocyte migration and survival, which are both parts of inflammatory leukocyte activation in IBD.

To obtain detailed information regarding leukocyte activation, we utilized the RAW264.7 macrophage cell line. Cells were treated with LPS+IFN-γ to induce inflammatory M1 activation (60, 61), which is characterized by increased MIF production (62, 63). Studies have shown LPS also activates HIF-1α mRNA transcription in monocytes and macrophages (64, 65) and HIF-1α regulates M1 polarization (66). Correspondingly to published literature, we observed a marked increase in HIF-1α mRNA (**Figure 5A**) and protein (**Figure 5B**) concentration in our model, which were reduced by MIF inhibition (**Figures 5A, B**). The profound role of MIF in the transcriptional activation of HIF-1α was likewise supported in literature (67, 68). However, in contrary to our findings, these studies did not identify MIF tautomerase as the key inducer of the HIF-1α transcription. In immune cells, HIF-1α can stimulate the gene transcription of numerous inflammatory cytokines, such as IL-6, TNF-α or CCL-2 (69–71). Since MIF regulates HIF-1α (**Figure 5**) and HIF-1α stimulates CCL2 and IL-6 mRNA transcription (**Figures 6A, B**) and protein expression (**Figures 7A, B**), MIF inhibition effectively counteracted these processes (**Figures 6A, B**). The surprising ineffectiveness of TE-11 on TNF-α mRNA transcription (**Figure 6C**) and protein expression (**Figure 7C**) suggests involvement of other MIF-independent transcription mechanisms. Consistent with other reports, our results demonstrated increased ROS production (72) (**Figure 7D**) and a self-protective transcriptional upregulation of the mitochondrial SOD2 antioxidant enzyme (73) in M1 macrophages (**Figure 6E**). In our model, TE-11 further increased SOD2 mRNA transcription (**Figure 6E**). HIF-1α can regulate SOD2 expression by directly binding and inhibiting the HRE element in the promoter of the SOD2 gene (74), inferring MIF tautomerase inhibition can reduce HIF-1α expression and in the absence of HIF-1α, SOD2 mRNA transcription is no longer impeded. Considering TE-11 has no direct ROS-scavenging activity (**Figure 1B**), putative upregulation of SOD2 may be one of the possible explanations for the slightly decreased ROS concentration when utilizing the MIF inhibitor (**Figure 7D**). Considering that we did not analyze SOD2 protein production in our study, this idea is merely hypothetical. In addition to oxidative stress, nitrosative stress can also be detected in active immune cells. In M1 macrophages, HIF-1α induces iNOS gene expression (75), which can be directly assessed by determining iNOS mRNA or indirectly, by measuring nitrite, the chemically more stable degradation product of NO. During the analysis of iNOS transcription, we found a marked increase in the iNOS mRNA level in LPS+IFN-γ-treated cells, however, we observed only a tendency in the reduction of iNOS transcription in response to TE-11 (**Figure 6D**). Since nitrite production was clearly declined following TE-11 treatment (**Figure 7E**) and TE-11 has no radical scavenging potential (**Figure 1B**), no further experiments were performed to reach statistical significance (**Figure 6B**). AMPK/PGC-1α/nuclear respiratory factor 1 (Nrf1) signaling pathway was found to protect mice in LPS-induced acute lung injury by improving mitochondrial function (76). Since we observed an improvement in mitochondrial bioenergetics in macrophages, as detailed later, we also analyzed Nrf1 mRNA transcription. Based on our results, we can assume that Nrf1 may not be involved in mitochondrial bioenergetic improvement in our model (**Figure 7E**).

Another important aspect of M1 polarization is metabolic reprogramming. LPS and IFN-γ treatment was shown to induce aerobic glycolysis in murine M1 cells (24, 77). To cite an instance, this metabolic switch can be induced by HIF-1α. HIF-1α increases the transcription of key transporters such as glucose transporter 1 and enzymes such as hexokinase II or pyruvate kinase M2 thereby activating glycolysis (78). This HIF-1α-induced glycolytic switch is essential to the inflammatory macrophage activation (79). We could also observe this metabolic switch in the M1 activated RAW264.7 cells (**Figure 8**). LPS+IFN-γ caused a significant increase in basal ECAR (**Figures 8A, B**), indicating a higher lactate production and enhanced glycolysis (80). The metabolic switch in the M1 cells was extremely pronounced. Oligomycin, an F_O_F_1_-ATPase inhibitor which reduces OXPHOS and mitochondrial ETC activation while increasing glycolysis failed to further increase ECAR in LPS+IFN-γ-treated cells (**Figure 8C**). However, it did further increase ECAR in the VEH and LPS+IFN-γ+TE-11-treated groups (**Figure 8C**). This finding suggests glycolysis proceeds at the highest possible flux among activated cells. In contrast, TE-11 intensely reduced glycolytic flux (**Figure 8B**). Overall, MIF tautomerase can likely control glycolysis via the MIF/HIF-1α axis. Metabolic reprogramming also involves the inhibition of the mitochondrial ETC and OXPHOS. Elevated NO concentration reduces the activity of pyruvate dehydrogenase and aconitase 2, which causes CAC corruption and suppresses mitochondrial ETC complexes (81). We found oxygen consumption was strongly reduced in cells with LPS+IFN-γ treatment when compared to the vehicle group. Neither oligomycin nor the mitochondrial uncoupler FCCP influenced OCR (**Figure 9A**), suggesting an intense inhibition of the mitochondrial ETC during M1 polarization. In contrast, in LPS+IFN-γ+TE-11-treated cells oligomycin reduced and FCCP improved OCR similarly as in VEH cells (**Figure 9A**), implying TE-11 may prevent the complete inhibition of ETC (**Figure 9A**). Changes in the determined parameters, such as basal respiration (**Figure 9B**), ATP production (**Figure 9C**), coupling efficiency (**Figure 9D**), maximal respiration (**Figure 9E**) and spare respiratory capacity (**Figure 9F**) reflect enhanced OXPHOS and improved mitochondrial energy production. Thus, MIF tautomerase inhibition reduced M1 activation associated metabolic reprogramming.

In our recent study, we used the mouse macrophage cell line RAW264.7. So, we must take it into account that human and mice macrophages may differ in many aspects. MIF and MIF receptors, however, are widely expressed in many human cells and organs (82), including monocytes (83) and macrophages (84), and inhibition of the MIF receptor CD74 prevents human M1 macrophage polarization (35). Since M1 activation with associated metabolic reprogramming is deeply involved in the pathomechanism of IBD, and MIF tautomerase is a pharmaceutical target for its inhibition, TE-11 may be a potential drug for future IBD treatment.

## 4 Materials and methods

### 4.1 Synthesis and purification of the test compound TE-11

The test compound (2*E*)-2-(pyridin-2-ylmethylidene)-3,4-dihydronaphthalen-1-one (TE-11), was synthesized at room temperature in ethanol as previously described (85). Purification was accomplished by recrystallization from methanol and by column chromatography. The structural characterization was performed based on Fourier-transform infrared spectroscopy methods and previously published NMR data. The compound was corroborated as E-isomer based on NMR measurements (86).

### 4.2 Isolation of peripheral blood polymorphonuclear leukocytes and eosinophil purification

All experiments involving primary cells of human peripheral blood were approved by the Institutional Review Board of the Medical University of Graz (EK 17–291 ex 05/06). Briefly, human peripheral blood polymorphonuclear leukocytes (PMNL) were isolated from citrated whole blood among healthy volunteers. Erythrocytes were removed by dextran sedimentation and PMNL were separated from peripheral blood mononuclear cells (PBMC) by density gradient centrifugation using PBMC spin medium (pluriSelect Life Science, Leipzig, DE). Eosinophils were further separated from neutrophils of the PMNL fraction by negative magnetic selection using MACS cell separation system (Eosinophil Isolation Kit, Miltenyi Biotech, Bergisch Gladbach, DE) with a resulting purity typically at ≥ 98%.

### 4.3 Chemotaxis assay

Chemotaxis assays were performed in a micro-Boyden chamber as previously described (47). Unless otherwise stated, all materials were procured from Merck (Darmstadt, DE). Eosinophil chemotaxis was performed with purified human eosinophils, whereas human PMNL preparations were used to assess the migratory responsiveness of neutrophils. Cells were resuspended in assay buffer (PBS with 0.9 mmol/L Ca^2+^ and 0.5 mmol/L Mg^2+^, supplemented with 0.1% BSA, 10 mmol/L HEPES, and 10 mmol/L glucose, pH 7.4), pretreated with TE-11 (20 µM) for 30 min at 37°C and allowed to migrate towards MIF (3 nM; Peprotech, London, UK; eosinophils: n = 7, neutrophils: n = 8), IL-8 (10 nM; Immunotools, Friesoythe, DE; neutrophils: n = 6) or CCL11 (10 nM; Immunotools, Friesoythe, DE; eosinophils: n = 7) for another 60 min at 37°C in a 48-well micro-Boyden chamber using PVP-free polycarbonate filters with a pore size of 5 µm (eosinophils) or 3 µm (neutrophils) (Sterlitech, Auburn, US). Migrating cells were enumerated by flow cytometry on a BD Canto II flow cytometer (acquisition set for 30 sec at medium flow rate). Eosinophils and neutrophils were gated by their forward and side scatter properties and by autofluorescence (**Supplementary Figure S1**). For all experiments, technical triplicates have been performed.

### 4.4 Apoptosis assay

Apoptosis assays were performed as previously described using an Annexin-V/Propidium iodide-based staining protocol (47). Unless otherwise stated, all materials were procured from Merck (Darmstadt, DE). Isolated PMNL (n=4) and purified eosinophils (n=4) from healthy volunteers were pretreated with TE-11 (20 µM) for 60 min in RPMI 1640 supplemented with 3% FBS and 1% Penicillin/Streptomycin. Afterwards, cells were incubated with MIF in PBS (500 nM, Peprotech, London, UK) or PBS+BSA (vehicle control) for 24 hours. Cells were stained with APC-Annexin-V (1/100) at 4°C in the dark for 20 min prior to adding Propidium iodide (PI; 1/50). Samples were immediately analyzed on a BD Canto II flow cytometer (acquisition set for 60 sec at medium flow rate). Total cell numbers of live cells (annexin-V negative/PI negative), early apoptotic cells (annexin-V positive/PI negative), late apoptotic cells (annexin-V positive/PI positive) and necrotic cells (annexin-V negative/PI positive) were recorded. For all experiments, technical triplicates were performed.

### 4.5 Animals

Male CD1 mice were bred and maintained at the animal facility of the Department of Biochemistry and Medical Chemistry, Medical School, University of Pécs. All experimental procedures were performed in full accordance with the European Communities Council Directive of 2010/63/EU under protocols approved by the Institutional Animal Use and Care Committee of the University of Pécs (Permit number: BA02/2000-65/2022). Animals were housed in temperature-controlled rooms on a 12/12 h light/dark cycle. Water and standard laboratory rodent chow were available ad libitum. At the time of TNBS-induction, the mice were 8-10 weeks old and organized randomly into vehicle (VEH), TNBS, and TNBS+TE-11 treatment groups. Body weight was matched, and 5 or 10 mg/kg body weight TE-11 was applied intraperitoneally as pretreatment (single injection) the day prior to TNBS treatment followed by daily administration for 3 days (**Figure 1C**). The VEH group received sterile PBS containing 5% DMSO. Following twelve hours fasting, mice were anesthetized with 5% isoflurane (Baxter Hungary Ltd, Budapest, Hungary) in 100% oxygen in an anesthetic chamber. TNBS (4 mg in 100 μl of 30% ethanol; Sigma-Aldrich, Missouri, USA) was administrated by single intracolonic injection (**Figure 1C**) through a catheter, which was carefully inserted into the colon approximately 3 cm deep. VEH group received equal amount of 30% ethanol. Animals were weighed daily throughout the experiment and sacrificed 72 hrs following TNBS administration (**Figure 1C**). Blood was collected; colons were extracted, weighted (g), length was measured (cm) and opened longitudinally to permit macroscopic evaluation of colon damage. Tissue samples were processed for further analysis.

### 4.6 Macroscopic scoring

To evaluate the tissue damage of the colon, we utilized a semiquantitative macroscopic scoring system formerly described by others (87). Briefly, individual scores were given in the presence of the following symptoms: 1. ulcers (0.5 points for each 0.5 cm); 2. adhesions (0 points: no adhesion, 1 point: 1 adhesion, 2 points: 2 or more adhesions or adhesions to other organs than the colon); 3. colon shortening (based on a mean length of a healthy colon, 1 point: >15%, 2 points: >25%); 4. wall thickness (mm); and 5. stool consistency and the presence of blood in the stool (haemorrhage, faecal blood, or diarrhea increase the total points by 1).

### 4.7 Colonic cytokine determination

In consideration of cytokine measurements, colon samples were weighed and homogenized in an extraction buffer containing protease inhibitor (Protease Inhibitor Cocktail (Sigma-Aldrich Missouri, USA), Tris (50 mmol/l), EDTA (10 mmol/l), and Triton X (1%) with a manual homogenizer (20 mg colon tissue/100 μl buffer). Samples were next centrifuged at 10,000 rpm for 10 minutes in which the protein concentration of the supernatants was determined via Bradford Reagent (Bio-Rad Laboratories). Following normalization of the protein content, IL-1β and IL-6 cytokines levels were determined by Mouse Uncoated (IL-1β and IL-6) ELISA kit (Invitrogen Waltham, Massachusetts, USA) in full accordance with the manufacturer’s guidance.

### 4.8 Measurement of radical scavenging effect

The direct free-radical scavenging activity of TE-11 was analyzed as formerly published (47). Briefly, we applied a cell free system using the Fenton reaction with 2 μM dihydrorhodamine 123 (DHR123) (Life Technologies, Carlsbad, CA, USA) fluorescent dye. Oxidation of DHR123 was induced by the reaction of 100 μM H_2_O_2_ (Sigma-Aldrich Missouri, USA) and 100 μM EDTA-Fe^2+^ salt in PBS. TE-11, resveratrol (Sigma-Aldrich, Missouri, USA) and tempol (Sigma-Aldrich, Missouri, USA) was diluted in PBS and applied in 20 μM final concentration. Fluorescent intensity (494 nm excitation and 517 nm emission) was measured immediately following the addition of DHR123 using FL6500 fluorescence spectrometer (Perkin-Elmer, Waltham, MA, USA).

### 4.9 Cell culture and treatments

In our cell culture experiments, we used RAW264.7 mouse monocyte/macrophage cell line (ECACC, Salisbury, UK). Cells were cultured in 5% CO_2_ at 37°C in endotoxin-tested Dulbecco’s Modified Eagle’s Medium (high glucose, 4.5 g/L, 2mM L-Glutamine; Biosera Cholet, France) and 10% FBS (Corning New York, USA) without addition of antibiotics. The day prior to the experiment cells were plated onto 24- or 96-well plates and cultured overnight. Next, the medium was replaced by a fresh one and cells were induced by 0.01 μg/ml IFN-γ (Merck Rahway, New Jersey, USA) and 0.1 μg/ml LPS (Sigma-Aldrich Missouri, USA). TE-11 was dissolved in DMSO (10 mM) and applied in 20 μM concentration as a pretreatment for 30 min prior to LPS and IFN-γ treatment. To exclude the effects of the vehicle, every cell received the precise amount of DMSO in 1:500 dilution.

### 4.10 ROS and nitrite production in macrophages

In the detection of reactive oxygen species and nitrite, we seeded RAW264.7 cells onto 96-well plates in a density of 10^5^ cells/well. Cells were treated with TE-11 (20 μM) as pretreatment and with LPS (0.1 μg/ml) and IFN-γ (0.01 μg/ml) for 24 hrs. Nitrite concentrations were measured by using two additional concentrations of TE-11 (5 µM, 10 µM) to show concentration-dependent effect. Following 24 hours of incubation, we utilized the same protocol for ROS and nitrite measurements as formerly described (47).

### 4.11 Cytokine production in macrophages

In consideration of cytokine concentration measurements, RAW264.7 cells were cultured in 24-well plates at a density of 5*10^5^ cells/well and treated with TE-11 as a pretreatment (20 μM) and with LPS (0.1 μg/ml) and IFN-γ (0.01 μg/ml) for 8 and 24 hours. TNF-α, IL-6, and CCL-2 levels were determined from the culturing media via Mouse (TNF-α, IL-6, CCL-2) uncoated ELISA kits (Invitrogen, Waltham, Massachusetts, USA). The concentration of IL-6 was measured by using two additional concentrations of TE-11 (5 µM, 10 µM) to demonstrate the concentration-dependent effect. HIF-1α was measured using a mouse HIF-1α ELISA Kit (FineTest, Wuhan, China). The supernatants from treated cells were collected, centrifuged to remove debris, and stored at -80°C until analysis. The ELISA protocol was achieved in full compliance to the manufacturer’s recommended protocol. Lastly, optical density was measured at 450 nm with Glomax Multi Detection System (Promega^®^, Madison, WI).

### 4.12 Measurement of mitochondrial bioenergetics

To analyze respiratory and glycolytic energy production, oxygen consumption rate (OCR) and extracellular acidification rate (ECAR) were determined by SeahorseXFp Analyzer (Agilent Technologies, Santa Clara, CA, USA). The day prior to the assay, the Seahorse XFp Sensor Cartridge was hydrated as suggested by the manufacturer’s protocol. The cells were plated at a starting density of 2*10^4^ cells/well onto Seahorse XFp Cell Culture Miniplates. RAW264.7 cells were induced with LPS (0.1 μg/ml) and IFN-γ (0.01 μg/ml) and treated with 20 µM TE-11 for 8 hours. OCR and ECAR measurements were performed by using two additional concentrations of TE-11 (5 µM, 10 µM) to demonstrate concentration-dependent effect. Following various treatments, the medium was replaced by Seahorse XF Base assay medium (pH 7.4) supplemented with 10 mM glucose, 2 mM L-glutamine, and 1 mM pyruvate. XFp Mito Stress Test Kit (Agilent Technologies, Santa Clara, CA, USA) was used for measuring mitochondrial function. The following modulators were injected sequentially: oligomycin, carbonyl cyanide 4-(trifluoromethoxy)phenylhydrazone (FCCP) and rotenone/antimycin A (Agilent Technologies, Santa Clara, CA, USA). The final concentration of the modulators was 1 μM. Bioenergetic parameters: basal respiration, ATP production, maximal respiration, spare respiratory capacity, non-mitochondrial respiration, proton leakage and coupling efficiency all were determined as formerly described (88).

### 4.13 RNA isolation and qPCR

RAW264.7 cells were plated on 24 well plates at a density of 5*10^5^ cells/well, pretreated with TE-11 (20 µM), and cultured with LPS (0.1 µg/mL) + IFN-γ (0.01 µg/mL) for 24 h. Total RNA was extracted from RAW 264.7 cells forming a confluent monolayer using MRX-03 MagCore^®^ triXact RNA Kit 631 (RBC Bioscience, Taiwan) in full accordance with the manufacturer’s protocol. Extracted RNA was quantified using Nanodrop 2000c spectrophotometer and Qubit 2.0 fluorometer (Thermo Fischer Scientific, Waltham, MA, USA). Two μg of total RNA was reverse-transcribed with M-MuLV RT (Maxima First Strand cDNA Synthesis Kit, Thermo Fischer Scientific, Waltham, MA, USA). Fifty ng cDNA was used in 10 μL final volume for real-time PCR using the Xceed qPCR SG 2× Mix (Institute of Applied Biotechnologies, Praha-Strašnice, Czech Republic) and a CFX384 Touch Real-Time PCR Detection System (Bio-Rad, Hercules, CA, USA). Data were analyzed using the ΔCt method. Primers were received from Integrated DNA Technologies (Belgium), except for Tnf (Invitrogen, Thermo Fisher Scientific, USA). The mRNA expression of the reference gene Ribosomal protein L27 was used for normalizing gene expression. Primers of gene expression include the following (**Table 1**):

**Table 1:** List of genes and primers for relative gene expression analysis.

Name	Accession NM	Sequence (5’-3’) (F-forward, R-reverse)	Amplicon size
Ribosomal protein L27 (Rpl27)	NM_011289	F-AGGTCAAGTTTGA GGAGCGATAC	141
		R-CCCACACAAATGC AATAGGCAG	
C-C motif chemokine ligand 2 (Ccl-2)	NM_011333	F-CTCAGCCAGATG CAGTTAACG	157
		R-CAGACCTCTCTCT TGAGCTTGG	
Hypoxia inducible factor 1, alpha subunit (Hif-1α)	NM_001422143	F-GCCACCAAGG AGGTACACAT	102
		R-AAGGAAGCCAT CACCAGCTTA	
Nuclear respiratory factor 1 (Nrf1)	NM_001164226	F-CGCTCATCCAG GTTGGTACA	74
		R-AGTGACTGTG GTTGGCAGTT	
Tumor necrosis factor (Tnf)	NM_001278601	F-ATGAGCACAGA AAGCATGATC	660
		R-TCACAGAGCA ATGACTCCAA	
Interleukin 6 (IL-6)	NM_031168	F-AGCCAGAGTCC TTCAGAGAGAT	108
		R-AGGAGAGCATT GGAAATTGGGG	
Nitric oxide synthase 2, inducible (Nos2)	NM_010927	F-GGGCAGCCTG TGAGACCTT	72
		R-CATTGGAAGT GAAGCGTTTCG	
Superoxide dismutase 2, mitochondrial (Sod2)	NM_013671	F-GGAGCAAGGT CGCTTACAGA	74
		R-GCGGAATAAGG CCTGTTGTT	

### 4.14 Statistical analyses

All statistical analyses were performed using SPSS version 28.0 statistics software (IBM, New York, USA). First, the normality of data distribution was investigated by Q-Q plot and/or box-plot, including the Shapiro–Wilk test. One-way ANOVA or Welch’s ANOVA with the appropriate *post hoc* tests were used to compare the means of groups. For establishing statistical significance between groups, partial eta squared value was calculated to demonstrate the effect size. Effect sizes ANOVA test was defined as small, when the η2 was between 0.01 and 0.06; moderate when η2 was between 0.06 and 0.14 and large when the rank η2 was greater than 0.14. Kruskal-Wallis non-parametric one-way ANOVA for independent samples with multiple pairwise comparisons was utilized to determine differences without the assumption of normality. The effect size for Kruskal-Wallis H test was regarded as small when the rank η2 was between 0.01-0.06; moderate when rank η2 was between 0.06-0.14 and large when the rank η2 was greater than 0.14. The paired samples t-test was used to determine differences between two groups. Effect sizes for paired sample t-test were classed as very small when the Cohen’s d value was less than 0.2; small when the d value was between 0.2-0.5; moderate when d was between 0.5-0.8 and large when the d was greater than 0.8. The p-values less than 0.05 were considered to be significant.

## Data Availability

The raw data supporting the conclusions of this article will be made available by the authors, without undue reservation.
