# Supplementary figures and images for: MIF tautomerase inhibitor TE-11 prevents inflammatory macrophage activation and glycolytic reprogramming while reducing leukocyte migration and improving Crohn’s disease-like colitis in male mice
Source: Front Immunol. 2025 Apr 22;16:1558079. doi: 10.3389/fimmu.2025.1558079 (PMC12053165; doi:10.3389/fimmu.2025.1558079)

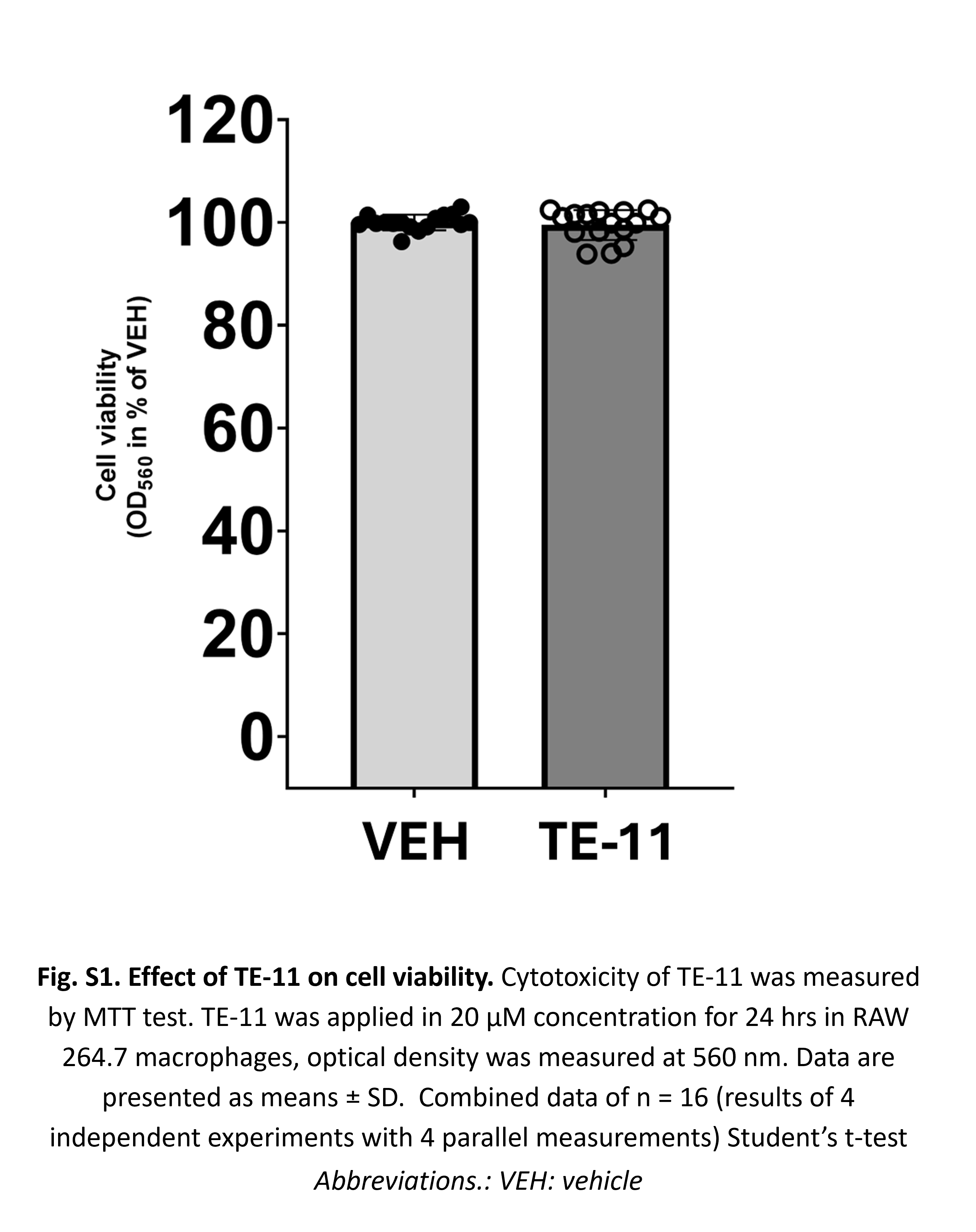

Supplement: Supplementary file 1 [file Image1.tif]

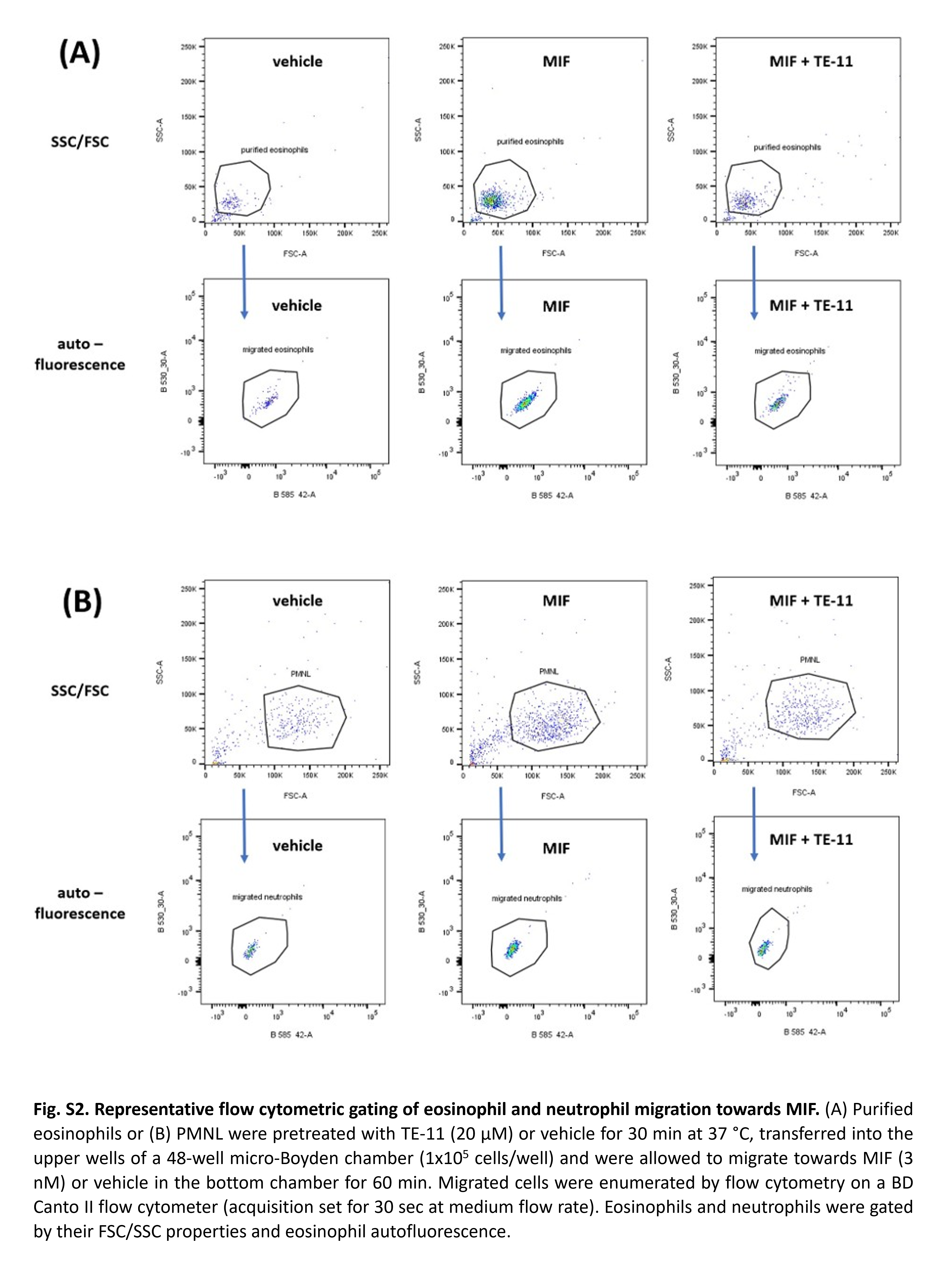

Supplement: Supplementary file 2 [file Image2.tif]

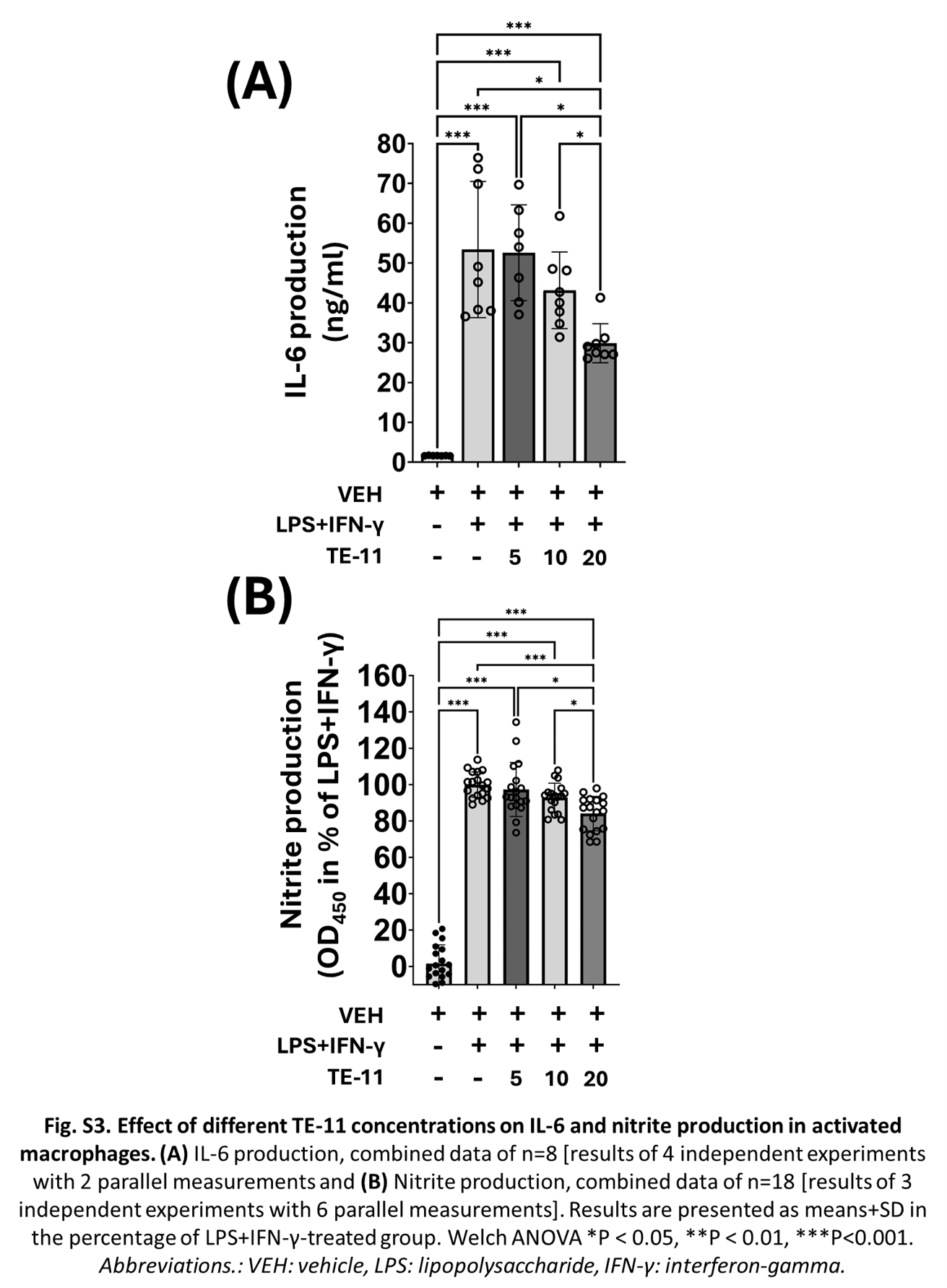

Supplement: Supplementary file 3 [file Image3.tif]

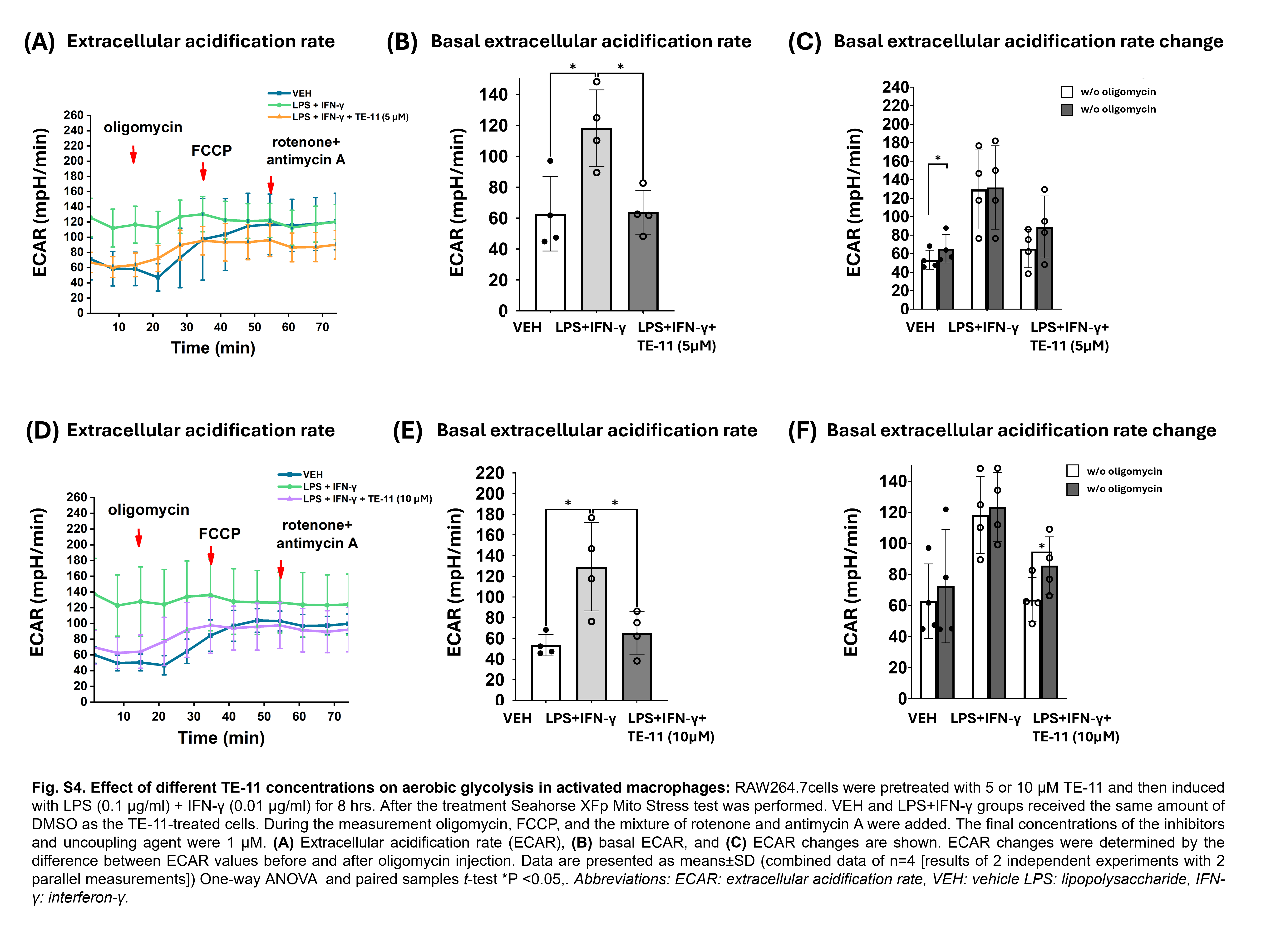

Supplement: Supplementary file 4 [file Image4.tif]

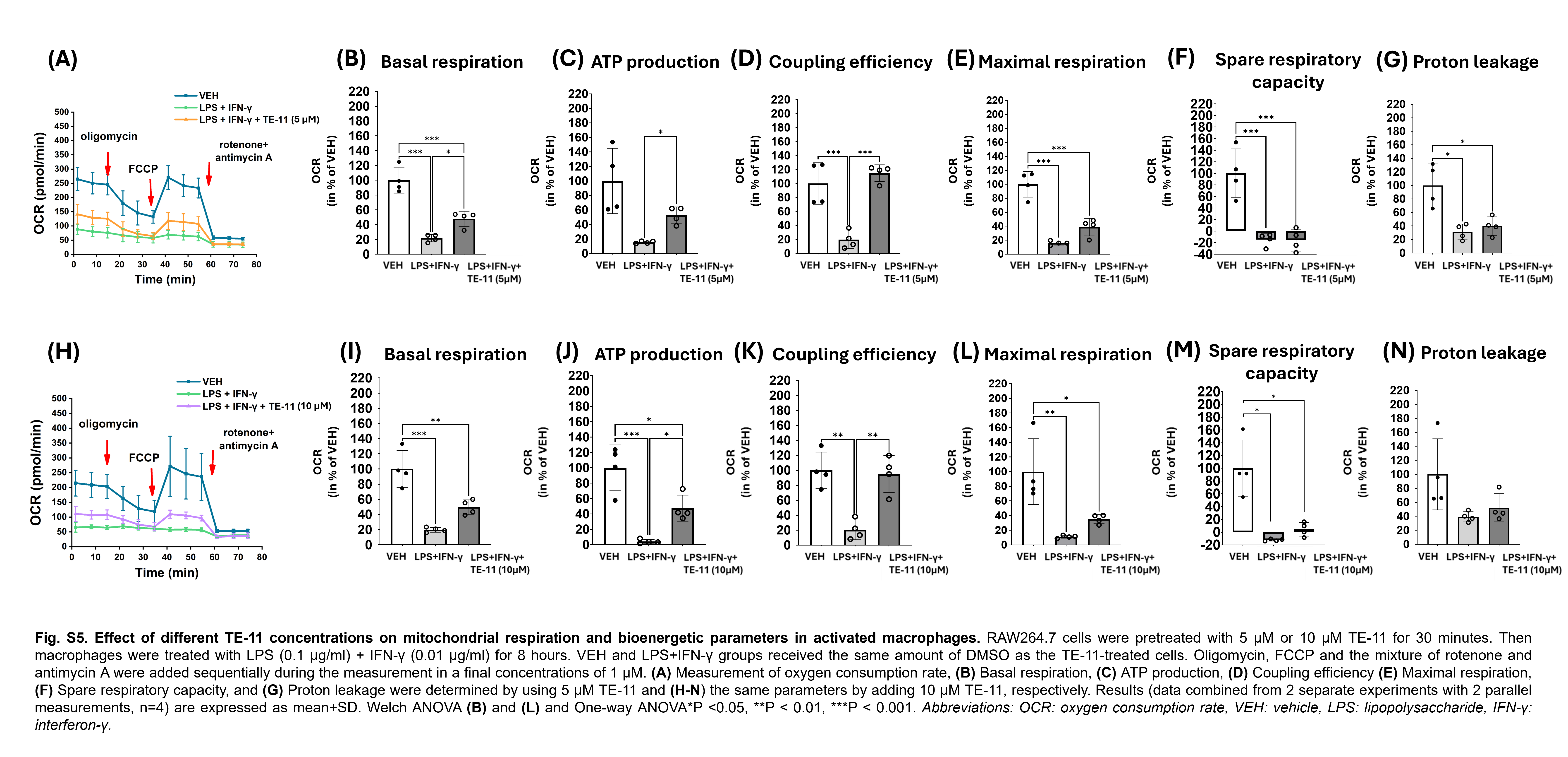

Supplement: Supplementary file 5 [file Image5.tif]
